# Supplementary material for: Critical Computational Evidence Regarding the Long-Standing Controversy over the Main Electrophilic Species in Hypochlorous Acid Solution
Source: Molecules. 2022 Mar 11;27(6):1843. doi: 10.3390/molecules27061843 (PMC8952510; doi:10.3390/molecules27061843)
Supplement: Supplementary file 1 [file molecules-27-01843-s001.zip › molecules-1586223-supplementary.pdf]

Supporting Information

Critical Computational Evidence for the Long-Term Controversy over the Main Electrophilic Species in Hypochlorous Acid Solution

Ke-Wei Chen [1], Yun-Dong Wu [1,2] and Tian-Yu Sun [2,*]

1. State Key Laboratory of Chemical Oncogenomics, School of Chemical Biology and Biotechnology, Peking University Shenzhen Graduate School, Shenzhen 518055, China
2. Shenzhen Bay Laboratory, Shenzhen 518132, China

E-mail: Tian-Yu_Sun@pku.edu.cn

Table of contents

Figure S1. Schematic diagram of flexible scanning of halogen or oxygen in HOX with C=C double bonds. S1

Figure S2. Select structures located for the transition state (TS 1-HOCl) of the electrophilic addition of trans-1,2-dimethyl ethylene and HOCl. S4

Figure S3. Select structures located for the transition state (TS-tri) of the electrophilic addition of trimethylethylene and HOCl S5

Figure S4. Schematic diagram of the potential energy surface of the reaction of OH or Cl radicals attacking trans-1,2-dimethyl ethylene S6

Figure S5. The potential energy surface for the reaction of different hypohalous acids with trans-1,2-dimethyl ethylenes S7

Figure S6. Select structures located for the transition state (TS 1-Cl_2) of the electrophilic addition of trans-1,2-dimethyl ethylene and Cl_2 .... S8

Figure S7. Select structures located for the transition state (TS 1-Cl_2O) of the electrophilic addition of trans-1,2-dimethyl ethylene and Cl_2O S9

Figure S8. Free energies for the reaction of different electrophiles (HOCl, Cl_2 and Cl_2O) with trans-1,2-dimethyl ethylene from the MP2/aug-cc-pVTZ method. S10

Cartesian Coordinates .... S**Error! Bookmark not defined.**

Figure S1. Schematic diagram of flexible scanning of halogen or oxygen in HOX with C=C double bonds.

In order to better understand whether the -Cl group or the -OH group in hypochlorous acid is the main electrophilic species, flexible scanning is used to find possible transition state structures. The computations show that the potential energy surface obtained by scanning the C-X bond did not find a saddle-like transition state structure for dimethyl ethylene and trimethyl ethylene. However, there is a saddle-like transition state structure for the potential energy surface of the C-O bond scanning. All calculations are performed under the M062X-D3 [35,36]/aug-cc-pVTZ [38,39,40] level of theory. Since the iodine atom needs to use a pseudopotential basis set, def2-TZVP was used [60].

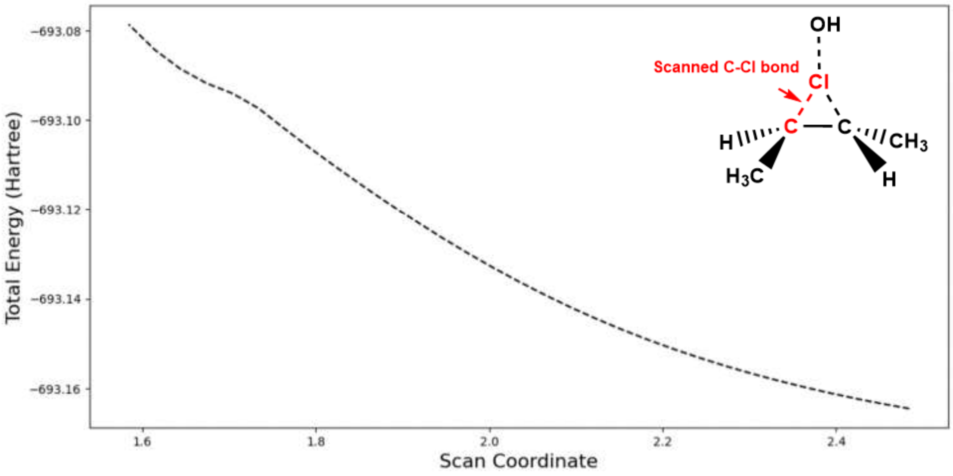

Figure S1a. Schematic diagram of flexible scanning of Cl in HOCl with trans-1,2-dimethyl ethylene.

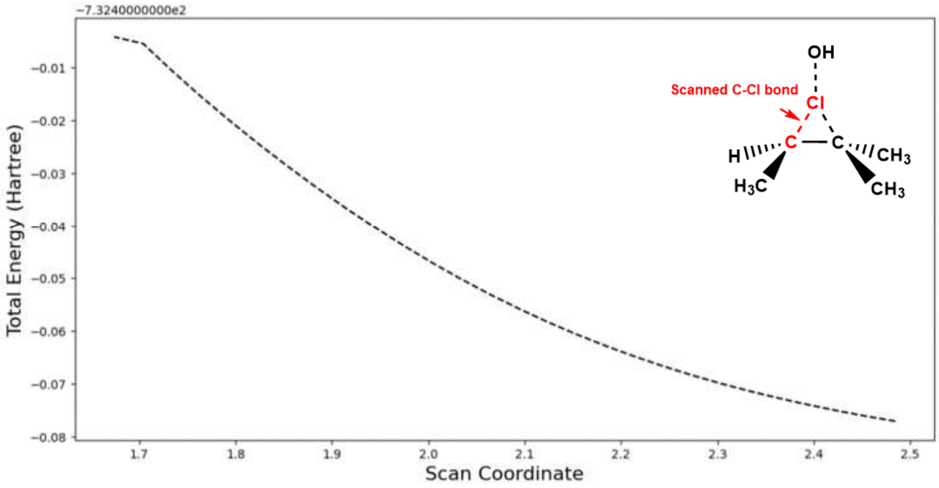

Figure S1b. Schematic diagram of flexible scanning of Cl in HOCl with trimethyl ethylene.

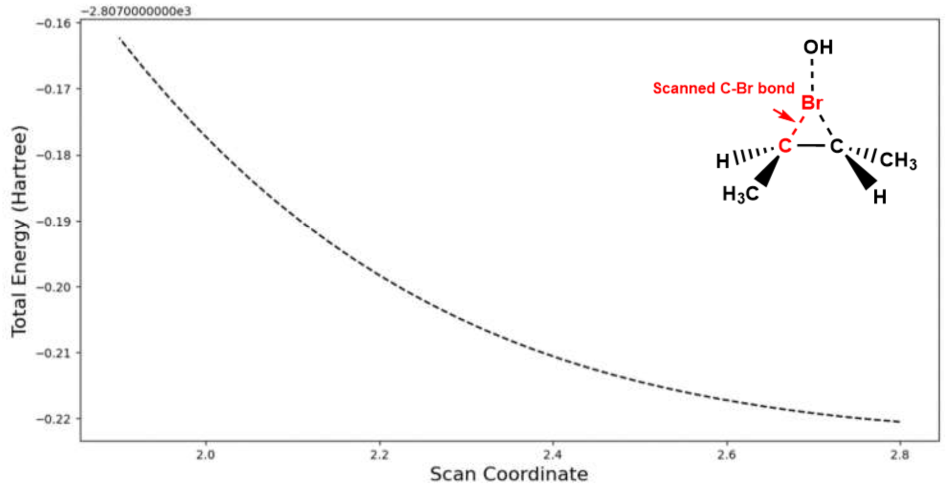

Figure S1c. Schematic diagram of flexible scanning of Br in HOBr with trans-1,2-dimethyl ethylene.

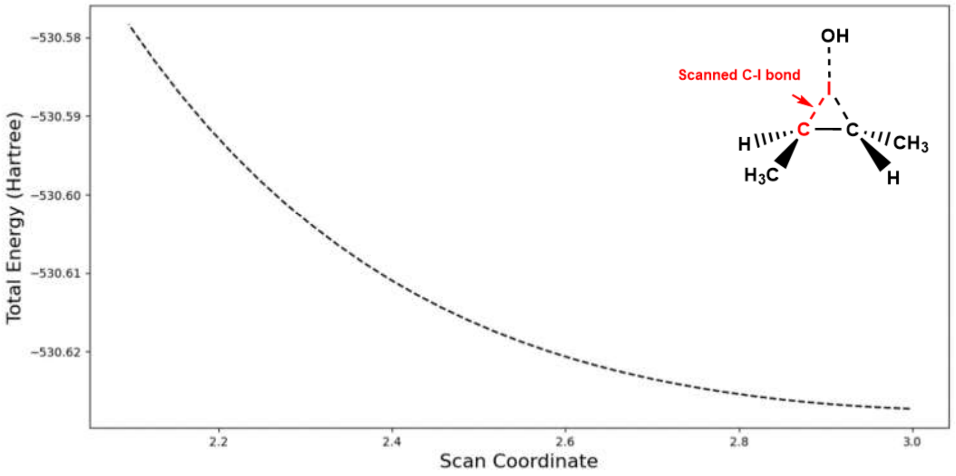

Figure S1d. Schematic diagram of flexible scanning of I in HOI with trans-1,2-dimethyl ethylene.

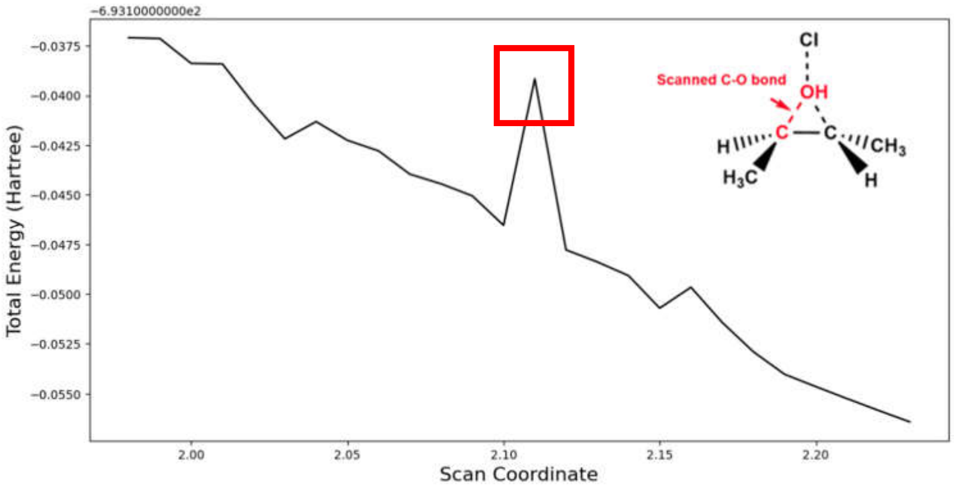

Figure S1e. Schematic diagram of flexible scanning of OH in HOCl with trans-1,2-dimethyl ethylene.

.

The transition state 3D structures for the electrophilic addition between the HOCl molecule and trans-1,2-dimethyl ethylene are demonstrated under different calculation conditions. The -OH group in the HOCl molecule adds to the ethylenes.

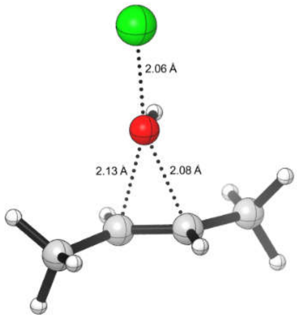

M062X-D3/aug-cc-pVTZ

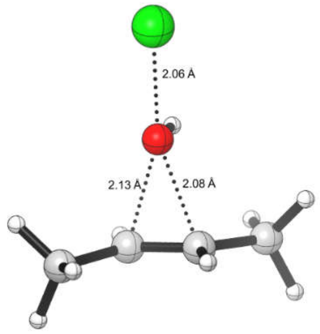

M062X-D3/def2-TZVPD

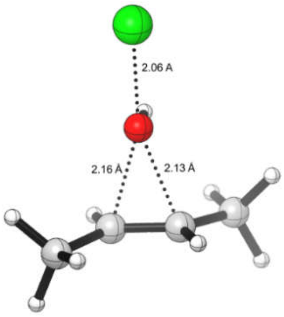

*ω*B97X-D/aug-cc-pVTZ

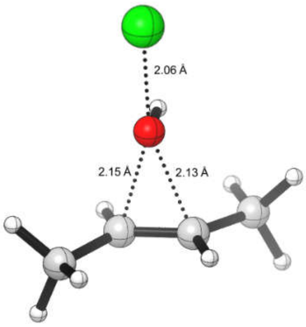

*ω*B97X -D/def2-TZVPD

Figure S2. Select structures located for the transition state (TS 1-HOCl) of the electrophilic addition of trans-1,2-dimethyl ethylene and HOCl**,** calculated at the M062X-D3 [35,36] and *ω*B97X -D [37] levels of theory, respectively. aug-cc-pVTZ [38,39,40] and def2-TZVPD [60] were used. C-O and O-Cl bond lengths (dashed lines) are given in Å and these structures were drawn using CYLview software [61].

The transition state 3D structures for the electrophilic addition between the HOCl molecule and trimethylethylene are demonstrated under different calculation conditions. The -OH group in the HOCl molecule adds to the ethylenes., These transition state structures will lead to the anti-Markovian rules' product, which is inconsistent with the experiments.

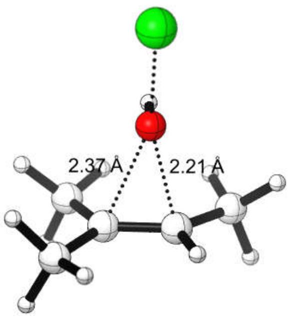

B3LYP-D3BJ/aug-cc-pVTZ

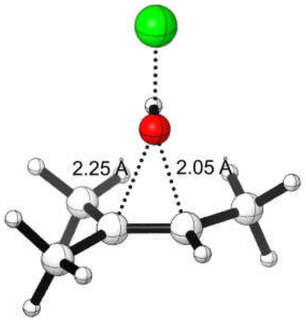

M062X-D3/aug-cc-pVTZ

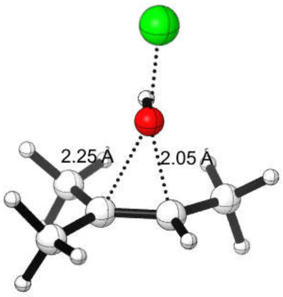

M062X-D3/def2-TZVPD

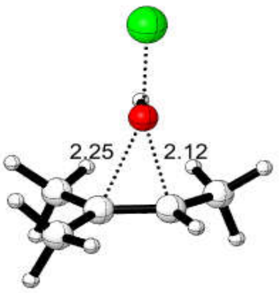

ωB97X-D/aug-cc-pVTZ

Figure S3. Select structures located for the transition state (TS-tri) of the electrophilic addition of trimethylethylene and HOCl, calculated at the B3LYP-D3BJ/aug-cc-pVTZ [62], M062X-D3/aug-cc-pVTZ, *ω*B97X-D/aug-cc-pVTZ and M062X-D3/def2-TZVPD levels of theory, respectively. C-O bond lengths (dashed lines) are given in Å.

The electrophilic addition of HOCl to olefins is not a free radical reaction. Among them, the structure of transition state TS 1-Cl-radical and TS 1-OH-radical cannot be located, and the free radical intermediates are not stable (> 25 kcal/mol).

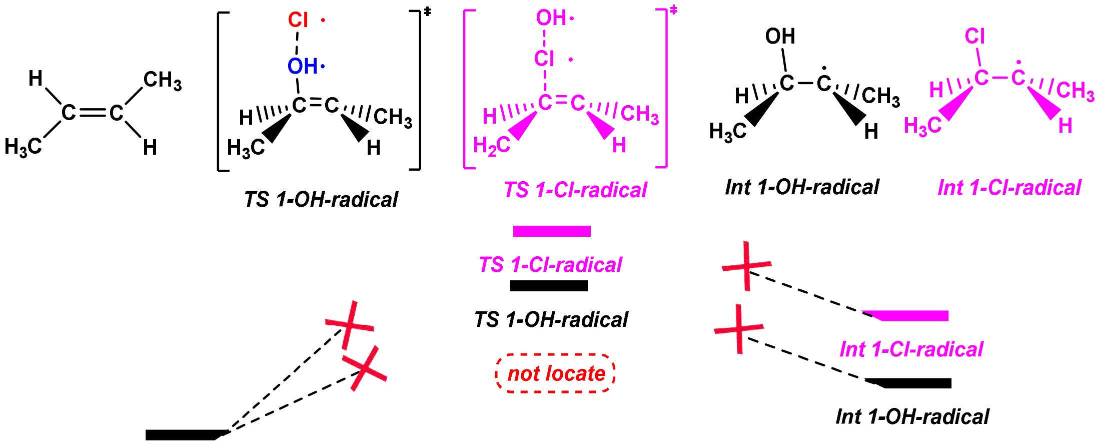

	Int 1-Cl-radical	Int 1-OH-radical
M062X-D3	30.4	26.3
WB97XD	28.2	25.0
DLPNO–CCSD(T)	33.0	28.3

Figure S4. Schematic diagram of the potential energy surface of the reaction of OH or Cl radicals attacking trans-1,2-dimethyl ethylene. The free energies to generate Int 1-Cl-radical and Int 1-OH-radical by different methods are included (in kcal mol^{-1}).

The M062X-D3/def2-TZVPD method was used to calculate the electrophilic addition reaction of different hypohalous acids with trans-1,2-dimethyl ethylene, and the results obtained are the same as those of M062X-D3/aug-cc-pVTZ mentioned in the text.

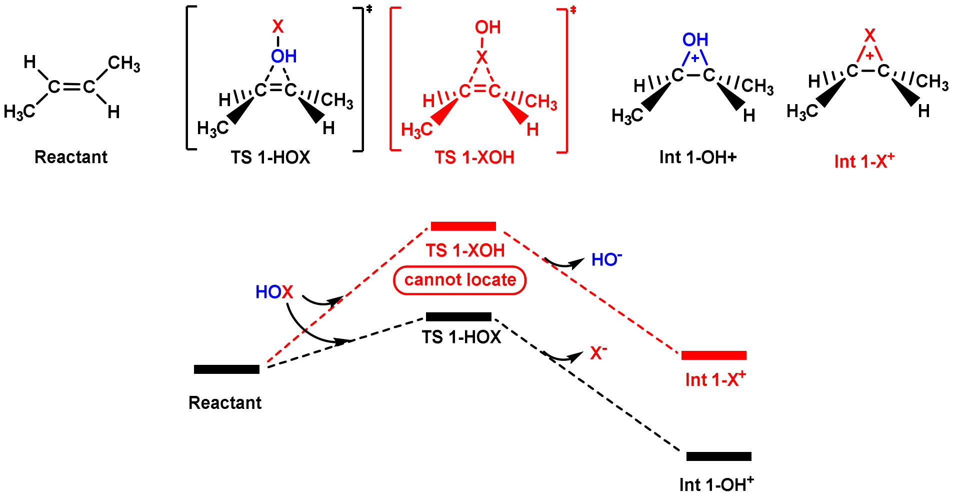

HOX	TS 1-HOX	Int 1-OH$^+$	Int 1-X$^+$
HOF	19.6	-44.2	-7.0
HOCl	31.6	-20.9	15.2
HOBr	32.8	-10.8	19.1
HOI	39.0	-8.9	23.0

Figure S5. The potential energy surface for the reaction of different hypohalous acids with trans-1,2-dimethyl ethylenes, calculated at the M062X-D3/def2-TZVPD level of theory. The Gibbs free energy of TS 1-HOX, Int 1-OH$^+$ and Int 1-X$^+$ are included (in kcal mol^{-1}).

The transition state 3D structures for the electrophilic addition between the Cl_2 molecule and trans-1,2-dimethyl ethylene are demonstrated under different calculation conditions, and the results are consistent with each other.

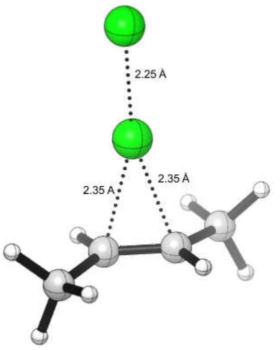

M062X-D3/aug-cc-pVTZ

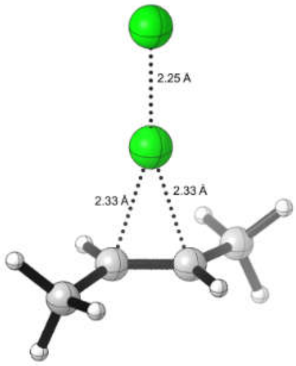

M062X-D3/def2-TZVPD

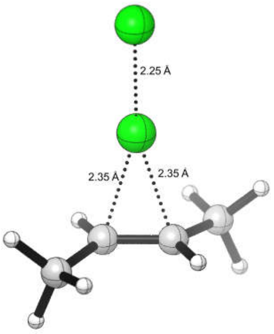

*ω*B97X-D/aug-cc-pVTZ

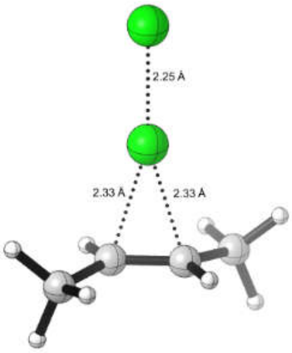

*ω*B97X-D/def2-TZVPD

Figure S6. Select structures located for the transition state (TS 1-Cl_2) of the electrophilic addition of trans-1,2-dimethyl ethylene and Cl_2. C-Cl bond lengths (dashed lines) are given in Å.

The transition state 3D structures for the electrophilic addition between the Cl_2O molecule and trans-1,2-dimethyl ethylene are demonstrated under different calculation conditions, and the results are consistent with each other.

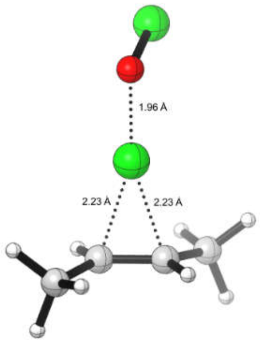

M062X-D3/aug-cc-pVTZ

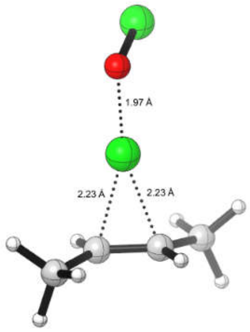

M062X-D3/def2-TZVPD

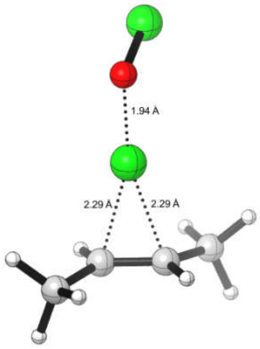

ωB97X -D/aug-cc-pVTZ

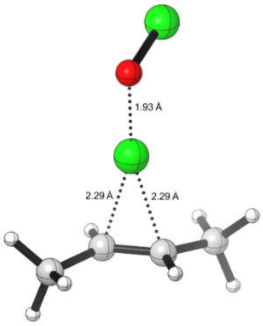

ωB97X -D/def2-TZVPD

Figure S7. Select structures located for the transition state (TS 1-Cl_2O) of the electrophilic addition of trans-1,2-dimethyl ethylene and Cl_2O. C-Cl bond and O-Cl bond lengths (dashed lines) are given in Å.

The MP2/aug-cc-pVTZ method was used to calculate the electrophilic addition reaction of three electrophiles: Cl_2, Cl_2O and HOCl respectively with trans-1,2-dimethyl ethylene, and the results obtained are the same as those by M062X-D3/aug-cc-pVTZ method in the main text.

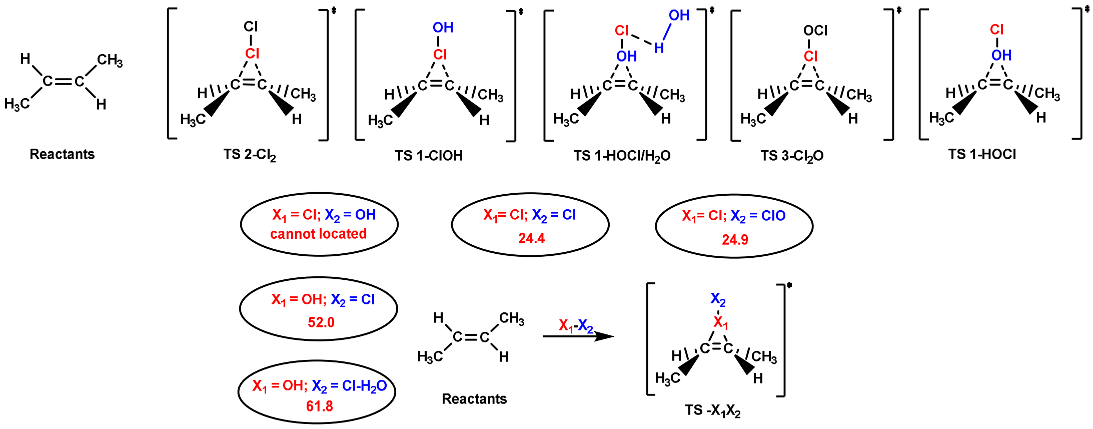

Figure S8. Free energies (in kcal mol^{-1}) for the reaction of different electrophiles (HOCl, Cl_2 and Cl_2O) with trans-1,2-dimethyl ethylene by the MP2/aug-cc-pVTZ method [63].

Cartesian Coordinates(fig. S2):

M062X-D3/aug-cc-pVTZ

C	-1.49273700	-0.55041200	0.39699900
C	-1.43600200	0.54484500	-0.41446100
H	-1.41655100	0.38029100	-1.48626900
C	-1.56311400	-1.94964700	-0.09096000
C	-1.54875900	1.94923600	0.05919900
H	-1.36946500	-2.00560900	-1.16068400
H	-0.85118900	-2.58048600	0.44150300
H	-2.55888800	-2.35369300	0.10702900
H	-0.78229500	2.57450200	-0.39897000
H	-2.51654900	2.35607400	-0.24287500
H	-1.46800400	2.01092200	1.14372400
Cl	2.58895400	-0.01621600	-0.01529600
O	0.52846900	-0.02189700	-0.01575400
H	0.53342800	0.49103400	0.80993900
H	-1.56678100	-0.38631800	1.46801200

M062X-D3/def2-TZVPD

C	-1.43442700	-0.54474600	0.41495600
C	-1.49210800	0.55114500	-0.39697700
H	-1.56849200	0.38732100	-1.46872300
C	-1.55108600	-1.94894700	-0.05872600
C	-1.56312600	1.95024800	0.09166400
H	-1.47297800	-2.01155100	-1.14417500
H	-0.78509400	-2.57686500	0.39867000
H	-2.52018500	-2.35313200	0.24604200
H	-2.56071700	2.35278700	-0.10536700
H	-1.36837700	2.00691800	1.16195400
H	-0.85276900	2.58299500	-0.44238100
Cl	2.58988400	0.01589500	0.01585400
O	0.52819200	0.02105000	0.01316700
H	0.53283400	-0.49292200	-0.81399100
H	-1.41331100	-0.38035200	1.48762100

ωB97X -D/aug-cc-pVTZ

C	-1.46361100	-0.53871400	0.41172900
C	-1.49076200	0.55835300	-0.39557600
H	-1.56051200	0.40068100	-1.46721900
C	-1.59887300	-1.94029600	-0.06107700
C	-1.56425700	1.95810000	0.08956000
H	-1.51242000	-2.01131300	-1.14466800
H	-0.84933700	-2.58078300	0.40447900
H	-2.57655700	-2.32714300	0.23644800
H	-2.55819900	2.36086000	-0.12112500
H	-1.38348100	2.02235800	1.16131800
H	-0.84797100	2.58909900	-0.43701900
Cl	2.61457600	0.00187700	0.01576800
O	0.55628800	0.02188700	0.01779900
H	0.54744400	-0.50546000	-0.79455000
H	-1.45203200	-0.37994400	1.48407100

ωB97X -D/def2-TZVPD

C	-1.46215300	-0.53885000	0.41201200
C	-1.48917000	0.55910700	-0.39549200
H	-1.56014800	0.40151900	-1.46820600
C	-1.60042600	-1.93995600	-0.06080600
C	-1.56433200	1.95846600	0.09003100
H	-1.51531700	-2.01156900	-1.14557400
H	-0.85141800	-2.58300600	0.40490000
H	-2.57981400	-2.32480300	0.23829600
H	-2.56035000	2.35961200	-0.12042500
H	-1.38322000	2.02329200	1.16283200
H	-0.84918100	2.59185200	-0.43768100
Cl	2.61571400	0.00174900	0.01629000
O	0.55398300	0.02119200	0.01571500
H	0.54733000	-0.50869800	-0.79676000
H	-1.45039700	-0.38006000	1.48549300

Cartesian Coordinates(fig. S3):

M062X-D3/aug-cc-pVTZ- TS-tri:

C	-1.40392400	-0.40016700	-0.05660600
C	-1.03309600	0.75054200	-0.70214700
H	-0.86569100	0.67008700	-1.76973300
C	-1.79451600	-0.46040400	1.38085100
C	-1.48535500	-1.68851200	-0.80188600
C	-1.06544200	2.13615200	-0.15387000
H	-1.13911500	-1.58752600	-1.82804900
H	-0.89941400	-2.45667800	-0.29406700
H	-2.52203600	-2.03552100	-0.80943600
H	-1.59869600	0.45975300	1.92370000
H	-2.86452500	-0.67776300	1.44363200
H	-1.27839900	-1.28664700	1.87271700
H	-1.99617900	2.62455400	-0.45248200
H	-1.00102600	2.16455300	0.93147600
H	-0.24590300	2.72099300	-0.56969900
Cl	2.82132900	-0.18292500	0.12585100
O	0.80580900	0.02000900	-0.16670900
H	0.69591900	0.32817900	0.74810500

M062X-D3/def2-TZVPD- TS-tri:

C	-1.40261900	-0.40130600	-0.05659600
C	-1.03092500	0.74989100	-0.70269700
H	-0.86181800	0.66905700	-1.77087100
C	-1.79785600	-0.45961500	1.37987700
C	-1.48368700	-1.68985800	-0.80197600
C	-1.06751300	2.13597100	-0.15559700
H	-1.13501800	-1.58938700	-1.82822000
H	-0.89994000	-2.45980600	-0.29264600
H	-2.52180500	-2.03528500	-0.81206700
H	-1.59608400	0.45904100	1.92477800
H	-2.87090000	-0.66748700	1.43880100
H	-1.29051000	-1.29121800	1.87351200
H	-2.00113300	2.62050700	-0.45485800
H	-1.00269300	2.16663400	0.93047400
H	-0.25012100	2.72413700	-0.57318900
Cl	2.82277200	-0.18221000	0.12592600
O	0.80536700	0.01993400	-0.16335100
H	0.69556900	0.33140300	0.75227600

B3LYP-D3BJ/aug-cc-pVTZ- TS-tri:

C	-1.42262600	-0.42882400	-0.05519200
C	-1.12428200	0.74138000	-0.69829000
H	-0.93497900	0.67366100	-1.76222000
C	-1.81656500	-0.51044000	1.38172600
C	-1.45316500	-1.71676100	-0.80867500
C	-1.22103300	2.12183900	-0.14862800
H	-1.11202300	-1.59874700	-1.83502300
H	-0.83999800	-2.46976100	-0.31022600
H	-2.47556000	-2.10610500	-0.82257100
H	-1.62530600	0.40449900	1.93531800
H	-2.88686600	-0.72997200	1.44610500
H	-1.29959400	-1.33777500	1.87032600
H	-2.15764800	2.58034700	-0.48093600
H	-1.20048900	2.15445600	0.93824700
H	-0.41230300	2.74302100	-0.53429300
Cl	2.89757200	-0.13323100	0.12065100
O	0.89486500	0.03824400	-0.15664200
H	0.75314700	0.40219900	0.73169400

ωB97X-D/aug-cc-pVTZ

C	-1.37495900	-0.43176600	-0.05022200
C	-1.10345600	0.74381200	-0.69535800
H	-0.94757600	0.68352700	-1.76550100
C	-1.74098300	-0.52436900	1.39290000
C	-1.42451300	-1.71401100	-0.81058300
C	-1.20228200	2.12071700	-0.13811500
H	-1.10156900	-1.59186600	-1.84235400
H	-0.80550000	-2.47049400	-0.32584800
H	-2.44956900	-2.09388500	-0.80668200
H	-1.53204100	0.38368000	1.95224800
H	-2.81094200	-0.73550500	1.47113500
H	-1.21872200	-1.35951600	1.86093400
H	-2.15363100	2.56501100	-0.44183300
H	-1.14834800	2.14987100	0.94805700
H	-0.41108800	2.74963100	-0.54562400
Cl	2.84184700	-0.13026900	0.11574200
O	0.82713300	0.03775300	-0.16955900
H	0.72767200	0.46579800	0.69260400

Cartesian Coordinates (fig. S4):

M062X-D3/Int 1-Cl-radical:

C	-1.10244300	0.55457100	0.34137100
C	0.17173600	0.49979100	-0.35721400
H	0.04624700	0.39054800	-1.43104600
C	1.15923000	1.57866700	0.00700100
H	2.10991100	1.43162800	-0.50053300
H	0.74858900	2.54341800	-0.29569100
H	1.32687100	1.59638500	1.08339900
Cl	0.99293400	-1.17593600	0.06947200
C	-2.27138100	-0.23606500	-0.10781700
H	-3.20879600	0.24710900	0.16769200
H	-2.25387600	-0.38958200	-1.18755300
H	-2.27625800	-1.22747900	0.36194100
H	-1.11541700	1.01709200	1.32072300

M062X-D3/Int 1-OH-radical:

C	-0.73883800	0.62909000	0.23712700
C	0.46980800	-0.01777800	-0.33908100
H	0.35817700	-0.09082300	-1.42463500
C	-2.09427100	0.11466400	-0.07963700
C	1.74682400	0.72397000	0.00045000
H	-2.37214500	-0.72525800	0.56905800
H	-2.85750900	0.88148200	0.05307500
H	-2.14252300	-0.25324000	-1.10702100
H	1.70151000	1.74782400	-0.37153300
H	1.88118700	0.75738900	1.08366000
H	2.60911000	0.22860700	-0.44387500
O	0.56391200	-1.40047500	0.08214500
H	0.61911400	-1.40218100	1.04576800
H	-0.60935300	1.36032500	1.02519600
H	-0.20278400	0.25948000	1.45822300
C	-0.60176800	1.82844400	0.02727000
H	-1.53926100	2.14594800	0.47869700
H	0.18833500	2.47701200	0.40596600
H	-0.67137100	1.94921600	-1.05455600
Cl	-1.72306900	-0.63236100	-0.09435700
C	1.30736000	-1.56211300	0.14251600
H	0.51704300	-2.27076700	-0.09245500
H	2.22105800	-1.88533500	-0.35380500
H	1.47406900	-1.56668700	1.22104100
H	0.76734100	-0.15185300	-1.39397900
O	2.05801700	0.66853900	-0.00266400
H	1.96359300	1.50647500	-0.46803000

Cartesian Coordinates(fig. S5):

M062X-D3/def2-TZVPD-TS 1-FOH:

C	1.00973700	0.55741500	0.40059100
C	1.01248500	-0.52980400	-0.40782200
C	1.10728200	-1.93735600	0.06715600
H	0.33260800	-2.55363800	-0.39317400
H	2.06983300	-2.36202900	-0.22897400
H	1.01871300	-1.99823100	1.15215900
C	1.05206100	1.96337200	-0.08066500
H	0.27374300	2.55852700	0.40007000
H	2.01080800	2.41648100	0.18420300
H	0.92771700	2.01507000	-1.16192600
O	-1.04877600	0.02704600	-0.05069000
H	-1.02173800	-0.52871100	0.74901800
F	-2.70822700	-0.01273200	-0.01835600
H	1.04564300	0.39656400	1.47534300
H	1.01752800	-0.36758300	-1.48155300

M062X-D3/def2-TZVPD-TS 1-ClOH:

C	1.49283100	0.55086100	0.39687000
C	1.43432700	-0.54500100	-0.41496600
C	1.55047400	-1.94918400	0.05892500
H	0.78384700	-2.57678600	-0.39784400
H	2.51913700	-2.35399300	-0.24636800
H	1.47297400	-2.01151700	1.14442300
C	1.56386300	1.95000700	-0.09155600
H	2.56121400	2.35283500	0.10634000
H	1.36984300	2.00697700	-1.16193900
H	0.85307300	2.58243500	0.44230700
O	-0.52833800	0.02115200	-0.01393200
H	-0.53289500	-0.49189200	0.81375700
H	1.41320600	-0.38083900	-1.48766000
H	1.56925400	0.38713000	1.46863900
Cl	-2.59011200	0.01625500	-0.01563800

M062X-D3/def2-TZVPD-TS 1-BrOH:

C	2.14140200	0.56533500	0.39796800
C	2.10659800	-0.53423500	-0.41699800
C	2.26157500	-1.93419000	0.05720000
H	1.52117600	-2.58640900	-0.40788400
H	3.24655600	-2.30602300	-0.23835900
H	2.17600400	-1.99963100	1.14193500
C	2.19918300	1.96402600	-0.09252800
H	3.19977600	2.36805700	0.08593400
H	1.98610700	2.01886300	-1.15942200
H	1.49688700	2.59518400	0.45322200
O	0.17157200	0.01088300	-0.00442800
H	0.14877900	-0.54514100	0.79365800
Br	-2.04890800	-0.00086600	-0.00820200
H	2.08645800	-0.36920600	-1.48931800
H	2.22490800	0.40192700	1.46890300

M062X-D3/def2-TZVPD-TS 1-IOH:

C	-2.76487200	-0.48945300	0.38181500
C	-2.44687300	0.58387600	-0.41435600
C	-2.52198900	1.99997500	0.04071000
H	-1.70695300	2.58648800	-0.38245100
H	-3.45892300	2.43981800	-0.31009300
H	-2.49232300	2.06776700	1.12829800
C	-2.91576200	-1.87591600	-0.11001300
H	-3.96060200	-2.18394300	0.00059600
H	-2.63248800	-1.96301000	-1.15778300
H	-2.32257600	-2.56757100	0.49122000
O	-0.63949800	-0.00115700	0.02347000
H	-0.63976000	0.30053000	0.95040100
I	1.72690400	-0.03940600	-0.00480400
H	-2.36907400	0.41078800	-1.48252800
H	-2.93025400	-0.30397500	1.44022800

M062X-D3/def2-TZVPD-Int 1-OH^+:

C	0.58192300	-0.21023800	0.42665200
C	-0.59895500	-0.14290400	-0.42672500
C	-1.97182200	-0.41037800	0.06632000
H	-2.69376800	0.21118700	-0.46096100
H	-2.19896900	-1.45711600	-0.14290500
H	-2.04149900	-0.23992200	1.13936900
C	1.94785400	-0.43370500	-0.11058100
H	2.01384600	-0.12222200	-1.15300300
H	2.68676600	0.09562400	0.48889400
H	2.15430100	-1.50340100	-0.04799700
O	-0.02060300	1.14648300	0.12634400
H	0.50024300	1.62253400	-0.55188800
H	-0.41975200	-0.19070200	-1.49411900
H	0.40965800	-0.40449600	1.47786300

M062X-D3/def2-TZVPD-Int 1-F^+:

C	-0.45214300	-0.57128500	-0.21106900
C	0.45214300	0.57128500	-0.21106900
C	0.00000000	1.95304700	-0.39726100
H	0.60405800	2.63727500	0.19556700
H	0.18009700	2.17681600	-1.45479600
H	-1.06101800	2.06410600	-0.18554200
C	0.00000000	-1.95304700	-0.39726100
H	1.06101800	-2.06410600	-0.18554200
H	-0.60405800	-2.63727500	0.19556700
H	-0.18009700	-2.17681600	-1.45479600
F	0.00000000	0.00000000	1.19170600
H	-1.51112200	-0.34678000	-0.26792000
H	1.51112200	0.34678000	-0.26792000

M062X-D3/def2-TZVPD-Int 1-Cl^+:

C	-0.45191000	-0.57047700	-0.48718800
C	0.45191000	0.57047700	-0.48718800
C	0.00000000	1.94627600	-0.78984500
H	0.61170500	2.67996300	-0.26932400
H	0.15676100	2.07443200	-1.86599200
H	-1.05568100	2.08574100	-0.56552700
C	0.00000000	-1.94627600	-0.78984500
H	1.05568100	-2.08574100	-0.56552700
H	-0.61170500	-2.67996300	-0.26932400
H	-0.15676100	-2.07443200	-1.86599200
Cl	0.00000000	0.00000000	1.28889600
H	1.50709500	0.33861200	-0.59257700
H	-1.50709500	-0.33861200	-0.59257700

M062X-D3/def2-TZVPD-Int 1-Br^+:

C	0.00000000	0.72473300	-0.92484000
C	0.00000000	-0.72473300	-0.92484000
C	1.20428800	-1.52124200	-1.24268600
H	1.18086100	-2.48913600	-0.74656300
H	1.15022900	-1.69841700	-2.32315900
H	2.12643900	-0.98744800	-1.02065400
C	-1.20428800	1.52124200	-1.24268600
H	-2.12643900	0.98744800	-1.02065400
H	-1.18086100	2.48913600	-0.74656300
H	-1.15022900	1.69841700	-2.32315900
Br	0.00000000	0.00000000	1.03596000
H	0.97045800	1.20106500	-1.03376800
H	-0.97045800	-1.20106500	-1.03376800

M062X-D3/def2-TZVPD-Int 1-I^+:

C	0.00000000	0.71738600	-1.25263400
C	0.00000000	-0.71738600	-1.25263400
C	1.20945100	-1.51304200	-1.59084100
H	1.19164400	-2.48538900	-1.10424200
H	1.17549000	-1.67349500	-2.67342700
H	2.12440400	-0.97870200	-1.34190200
C	-1.20945100	1.51304200	-1.59084100
H	-2.12440400	0.97870200	-1.34190200
H	-1.19164400	2.48538900	-1.10424200
H	-1.17549000	1.67349500	-2.67342700
I	0.00000000	0.00000000	0.88840500
H	-0.96967000	-1.19309700	-1.36230800
H	0.96967000	1.19309700	-1.36230800

Cartesian Coordinates(fig. S6):

M062X-D3/aug-cc-pVTZ

C	-1.70023100	0.55033700	-0.40386200
C	-1.70014000	-0.55045800	0.40383400
H	-1.73282700	-0.39025900	1.47698000
C	-1.81698700	-1.94835700	-0.08643900
H	-2.81935400	-2.31831400	0.14258500
H	-1.65966700	-2.00657100	-1.16196100
H	-1.10919100	-2.60130600	0.42344900
C	-1.81751300	1.94819800	0.08643900
H	-1.10984100	2.60137700	-0.42331200
H	-2.81995700	2.31789600	-0.14263000
H	-1.66031700	2.00638100	1.16198800
H	-1.73267600	0.39013400	-1.47701400
Cl	0.54785300	0.00000900	-0.00000500
Cl	2.79644400	0.00012800	0.00000900

M062X-D3/def2-TZVPD

C	1.68812800	-0.55115700	-0.40451600
C	1.68830200	0.55126800	0.40443100
H	1.72339500	0.39115700	1.47833300
C	1.81148100	1.94820800	-0.08685200
H	2.81842500	2.31045700	0.13897500
H	1.65115800	2.00779200	-1.16263000
H	1.11006800	2.60656900	0.42655500
C	1.81188900	-1.94806800	0.08677300
H	1.11052900	-2.60669000	-0.42635600
H	2.81887200	-2.31001600	-0.13931400
H	1.65189500	-2.00765100	1.16260200
H	1.72265100	-0.39101900	-1.47843100
Cl	-0.53938300	-0.00006500	0.00010000
Cl	-2.79036900	-0.00005900	-0.00002700

ωB97X -D/aug-cc-pVTZ

C	-1.69629200	-0.55207300	0.40177400
C	-1.69629300	0.55207600	-0.40177400
H	-1.73346200	0.39725600	-1.47489400
C	-1.83600700	1.94733400	0.08695000
H	-2.84725500	2.29389900	-0.14072900
H	-1.67867900	2.01726800	1.16192300
H	-1.14612500	2.61507600	-0.42836800
C	-1.83601700	-1.94733100	-0.08694600
H	-1.14612900	-2.61507400	0.42836200
H	-2.84726300	-2.29389400	0.14075100
H	-1.67870800	-2.01726800	-1.16192200
H	-1.73344900	-0.39725300	1.47489500
Cl	0.55701500	0.00000000	-0.00000400
Cl	2.80761600	-0.00000300	0.00000200

ωB97X -D/def2-TZVPD

C	1.68171100	-0.55299500	-0.40221200
C	1.68164100	0.55307800	0.40221600
H	1.72160300	0.39808300	1.47631700
C	1.82780300	1.94708800	-0.08727600
H	2.84331900	2.28657100	0.13817300
H	1.66853400	2.01817200	-1.16298800
H	1.14330000	2.62006000	0.43078100
C	1.82807200	-1.94701100	0.08728700
H	1.14393200	-2.62018500	-0.43096600
H	2.84374900	-2.28622200	-0.13780600
H	1.66847800	-2.01811300	1.16295100
H	1.72141500	-0.39796300	-1.47631600
Cl	-0.54651000	-0.00002100	-0.00003800
Cl	-2.79876600	-0.00005900	0.00002400

Cartesian Coordinates(fig. S7):

M062X-D3/aug-cc-pVTZ

C	-2.05721900	-0.29396700	0.52220700
C	-1.91784200	0.73880900	-0.37478300
H	-1.30291600	2.21670800	1.04229000
C	-2.60652400	-1.62950100	0.17368300
H	-2.04252100	-2.42308600	0.66176900
H	-3.63399800	-1.68559600	0.54135600
H	-2.61130500	-1.78794800	-0.90319800
H	-1.89972700	-0.07192500	1.57241600
Cl	0.05368400	-0.30041400	-0.21170800
O	1.92710700	-0.81955100	-0.50785700
Cl	2.95469800	0.22233100	0.28751600

M062X-D3/def2-TZVPD

C	-2.05015400	-0.29505300	0.52102700
C	-1.90904400	0.73871500	-0.37556200
H	-2.15518100	0.54123800	-1.41460000
C	-1.64565000	2.14768500	0.01543000
H	-0.91731300	2.60833800	-0.65210600
H	-2.57804800	2.71129900	-0.07912600
H	-1.29828200	2.21642900	1.04553900
C	-2.60199600	-1.62924500	0.17020100
H	-2.04114600	-2.42541500	0.65962100
H	-3.63126400	-1.68233500	0.53596700
H	-2.60537600	-1.78753400	-0.90752700
H	-1.89564100	-0.07396500	1.57281100
Cl	0.05338700	-0.29885600	-0.20953300
O	1.92147700	-0.81644500	-0.50290900
Cl	2.94611300	0.21949700	0.28459900

ωB97X -D/aug-cc-pVTZ

C	-2.08251500	-0.30262300	0.51961300
C	-1.95437100	0.72963400	-0.37000100
H	-2.19227600	0.53313800	-1.40974700
C	-1.71031600	2.14318800	0.01458700
H	-0.97989900	2.60887000	-0.64636600
H	-2.64573200	2.69704800	-0.09722400
H	-1.37738300	2.22944400	1.04754200
C	-2.61769200	-1.64474100	0.17566400
H	-2.04032700	-2.43306100	0.65752500
H	-3.63980500	-1.71533800	0.55588800
H	-2.63590500	-1.80893800	-0.90045600

H	-2.16386400	0.53959000	-1.41258200
C	-1.65165000	2.14765300	0.01352200
H	-0.92356800	2.60518700	-0.65503400
H	-2.58204200	2.71292300	-0.08066000
H	-1.92743100	-0.08569400	1.57084700
Cl	0.08779300	-0.29791200	-0.21638500
O	1.93710700	-0.79898700	-0.51607700
Cl	2.97875100	0.22812900	0.29352800

ωB97X -D/def2-TZVPD

C	-2.07720300	-0.30232100	0.51780500
C	-1.94665000	0.73060500	-0.37043100
H	-2.18408300	0.53592400	-1.41188100
C	-1.70195800	2.14365100	0.01623400
H	-0.96818500	2.60992900	-0.64260800
H	-2.63727200	2.69961900	-0.09784400
H	-1.37177200	2.22890100	1.05137200
C	-2.61308000	-1.64374400	0.17163100
H	-2.03534900	-2.43430100	0.65218400
H	-3.63617900	-1.71503900	0.55256700
H	-2.63212600	-1.80642800	-0.90587000
H	-1.92435700	-0.08668900	1.57087900
Cl	0.09070100	-0.29804600	-0.21407100
O	1.92893600	-0.79753100	-0.50764300
Cl	2.96760400	0.22623400	0.28941900

Cartesian Coordinates (fig. 8):

M062X-D3/aug-cc-pVTZ-Reactant:

C	0.53315600	0.39529400	-0.00009500
C	-0.53316300	-0.39568000	-0.00013200
H	-0.38113600	-1.47265300	-0.00005200
C	1.95126400	-0.07954000	0.00006500
C	-1.95127500	0.07977500	0.00006700
H	2.00183300	-1.16812400	-0.00041300
H	2.48804100	0.29101100	-0.87557000
H	2.48751100	0.29022300	0.87637500
H	-2.48760800	-0.28944300	0.87652700
H	-2.00128100	1.16837100	-0.00068500
H	-2.48818800	-0.29073400	-0.87546000
H	0.38094200	1.47225200	-0.00014500

M062X-D3/aug-cc-pVTZ-TS 1-Cl_2:

C	-1.70023100	0.55033700	-0.40386200
C	-1.70014000	-0.55045800	0.40383400
H	-1.73282700	-0.39025900	1.47698000
C	-1.81698700	-1.94835700	-0.08643900
H	-2.81935400	-2.31831400	0.14258500
H	-1.65966700	-2.00657100	-1.16196100
H	-1.10919100	-2.60130600	0.42344900
C	-1.81751300	1.94819800	0.08643900
H	-1.10984100	2.60137700	-0.42331200
H	-2.81995700	2.31789600	-0.14263000
H	-1.66031700	2.00638100	1.16198800
H	-1.73267600	0.39013400	-1.47701400
Cl	0.54785300	0.00000900	-0.00000500
Cl	2.79644400	0.00012800	0.00000900

M062X-D3/aug-cc-pVTZ-TS 1-Cl_2O:

C	-2.05721900	-0.29396700	0.52220700
C	-1.91784200	0.73880900	-0.37478300
H	-2.16386400	0.53959000	-1.41258200
C	-1.65165000	2.14765300	0.01352200
H	-0.92356800	2.60518700	-0.65503400
H	-2.58204200	2.71292300	-0.08066000
H	-1.30291600	2.21670800	1.04229000
C	-2.60652400	-1.62950100	0.17368300
H	-2.04252100	-2.42308600	0.66176900
H	-3.63399800	-1.68559600	0.54135600
H	-2.61130500	-1.78794800	-0.90319800
H	-1.89972700	-0.07192500	1.57241600
Cl	0.05368400	-0.30041400	-0.21170800
O	1.92710700	-0.81955100	-0.50785700
Cl	2.95469800	0.22233100	0.28751600

M062X-D3/aug-cc-pVTZ-Int 1-Cl^+:

C	0.45146500	0.57038000	-0.49160100
C	-0.45146500	-0.57038000	-0.49160100
H	-1.50585800	-0.33853300	-0.59341300
C	0.00000000	-1.94595500	-0.79141900
H	-0.61159200	-2.67786300	-0.27016200
H	-0.15677400	-2.07579500	-1.86655200
H	1.05488000	-2.08461400	-0.56701000
Cl	0.00000000	0.00000000	1.29356000
C	0.00000000	1.94595500	-0.79141900
H	0.61159200	2.67786300	-0.27016200
H	0.15677400	2.07579500	-1.86655200
H	-1.05488000	2.08461400	-0.56701000
H	1.50585800	0.33853300	-0.59341300

M062X-D3/aug-cc-pVTZ-TS 2-Cl^+-H_2O:

C	-0.12177300	-0.38495000	-0.01485100
C	-1.14690600	0.53784600	0.44847300
H	-1.38578800	0.45835800	1.50308400
C	-1.30429000	1.88168200	-0.17716700
H	-2.33044500	2.22618700	-0.07105800
H	-0.65547800	2.57199800	0.36300100
H	-1.02113600	1.86334300	-1.22775500
Cl	-2.19449100	-0.75282000	-0.40886300
C	0.39118800	-1.50430800	0.78588000
H	0.38695900	-2.41925600	0.19566700
H	1.43744000	-1.27930800	1.00627000
H	-0.15922500	-1.62902300	1.71431200
H	0.24917200	-0.23119900	-1.02081000
O	1.62888600	1.16241700	0.54552500
H	1.56523200	1.97396100	0.02972300
H	2.37039700	0.66255400	0.14706500
O	3.58941300	-0.42205800	-0.58222500
H	3.75055700	-0.18510500	-1.50224300
H	4.44295800	-0.31906100	-0.14699200

M062X-D3/aug-cc-pVTZ-Product:

C	0.94511900	-0.16701800	-0.31450200

C	-0.29904100	0.39041000	0.38209600
H	-0.20278400	0.25948000	1.45822300
C	-0.60176800	1.82844400	0.02727000
H	-1.53926100	2.14594800	0.47869700
H	0.18833500	2.47701200	0.40596600
H	-0.67137100	1.94921600	-1.05455600
Cl	-1.72306900	-0.63236100	-0.09435700
C	1.30736000	-1.56211300	0.14251600
H	0.51704300	-2.27076700	-0.09245500
H	2.22105800	-1.88533500	-0.35380500
H	1.47406900	-1.56668700	1.22104100
H	0.76734100	-0.15185300	-1.39397900
O	2.05801700	0.66853900	-0.00266400
H	1.96359300	1.50647500	-0.46803000
